# Osmotic behaviour of human mesenchymal stem cells: Implications for cryopreservation

**DOI:** 10.1371/journal.pone.0184180

**Published:** 2017-09-08

**Authors:** Elisa Casula, Gino P. Asuni, Valeria Sogos, Sarah Fadda, Francesco Delogu, Alberto Cincotti

**Affiliations:** 1 Dipartimento di Ingegneria Meccanica, Chimica e dei Materiali, Università degli Studi di Cagliari, Cagliari, Italy; 2 Centro Interdipartimentale di Ingegneria e Scienze Ambientali, Università degli Studi di Cagliari, Cagliari, Italy; 3 Dipartimento di Scienze Biomediche, Università degli Studi di Cagliari, Cittadella Universitaria, Monserrato, Italy; 4 Centre for Process Systems Engineering, Department of Chemical Engineering, Imperial College London, London, United Kingdom; Instituto Butantan, BRAZIL

## Abstract

Aimed at providing a contribution to the optimization of cryopreservation processes, the present work focuses on the osmotic behavior of human mesenchymal stem cells (hMSCs). Once isolated from the umbilical cord blood (UCB) of three different donors, hMSCs were characterized in terms of size distribution and their osmotic properties suitably evaluated through the exposure to hypertonic and isotonic aqueous solutions at three different temperatures. More specifically, inactive cell volume and cell permeability to water and di-methyl sulfoxide (DMSO) were measured, being cell size determined using impedance measurements under both equilibrium and dynamic conditions. Experimental findings indicate that positive cell volume excursions are limited by the apparent increase of inactive volume, which occurs during both the shrink-swell process following DMSO addition and the subsequent restoration of isotonic conditions in the presence of hypertonic solutions of impermeant or permeant solutes. Based on this evidence, hMSCs must be regarded as imperfect osmometers, and their osmotic behavior described within a scenario no longer compatible with the simple two-parameter model usually utilized in the literature. In this respect, the activation of mechano-sensitive ion-channels seemingly represents a reasonable hypothesis for rationalizing the observed osmotic behavior of hMSCs from UCB.

## Introduction

The progressive ageing of population in western and industrialised countries represents one of currently emerging demographic trends with straightforward implications for well-being and welfare policies. Involving the enhanced incidence of degenerative diseases, it lays a definite societal challenge on the development of regenerative medicine to contrast tissue degeneration. In this respect, stem cells seem to show the greatest promise.

The ability to readily expand in culture, while maintaining a self-renewing phenotype, makes hMSCs ideal for many cell-based therapies [1,2]. Abundant literature points out their capability of reproducibly differentiating to adipocytes, osteoblasts, and chondrocytes in vitro [3] and rescuing mesenchymal tissue disorders in humans [4]. In addition, in contrast with induced pluripotent and embryonic stem cells, adult hMSCs do not raise ethical issues. Hence, the beneficial impact on the authority approval and public acceptance of cell therapies in countries forbidding the isolation of embryonic stem cells.

Although bone marrow is the primary source of adult hMSCs, its invasive aspiration makes peripheral sources, such as UCB [5,6] and adipose tissue [7], preferable. However, contradictory reports on hMSC isolation from such sources [8,9] suggest a strong dependence on the experimental method utilized [2]. This circumstance clarifies the stringent need of preserving hMSCs, which is a crucial issue for the supply chain of regenerative medicine [10].

Affording long shelf lives, genetic stability, reduced microbial contamination risks, and cost effectiveness [10], freezing bio-specimens to cryogenic temperature is the main preservation method. Cryopreservation comprises four different stages entailing the cooling of cells to cryogenic temperatures in the presence of cryoprotectant agents (CPAs) and their storage as well as the thawing and subsequent restoration of physiological conditions for specific usages. Unfortunately, cells subjected to cryopreservation are not immune to damage [11]. Not only cooling and thawing can damage cells due to, e.g., intra-cellular ice formation, excessive solute concentration and cell volume excursions [10,11], but CPAs can be cytotoxic themselves, particularly at high concentration, long contact times, and relatively high temperature. The most widely used CPA, i.e. dimethyl sulfoxide (DMSO), is no exception.

Damage results in a loss of viable or functional cells up to 50% [12]. While such loss is acceptable for some cell lineages for research application, it becomes unacceptable in clinical practices, especially those involving hMSCs from UCB [13]. In principle, cell expansion/proliferation may solve the problem, but an increased number of passages would inexorably make these cells lose their inherent features [14]. In addition, it would raise production costs and require approval from regulatory bodies. This pushes biopharmaceutical companies to develop optimized cryopreservation processes with reduced loss of viable cells.

In this regard, it is worth noting that the number of experimental variables and parameters is large enough to preclude any systematic exploration of experimental processing conditions. Therefore, empirical measurement campaigns based on brute force approach can hardly represent a satisfactory strategy. Rather, beneficial clues to best practices in cryopreservation can be obtained by numerical simulation [10], which guarantees the identification of most influential factors and the drastic reduction of experimental efforts.

The present study aims at providing a contribution along this line. To this aim, the experimental investigation of the osmotic behaviour at three different temperatures of hMSCs from the UCB of three different donors is combined with the numerical description of the underlying processes. In particular, impedance measurements were performed to estimate cell size distribution and cell response to hypertonic conditions and subsequent isotonic condition restoration. Based on previous work modeling intracellular ice formation in a cell population with distributed size [15–17], hydraulic conductivity (L_*P*_), membrane permeability to DMSO (*P*_*CPA*_), and inactive cell volume (*υ*_*b*_) were determined using the 2-parameter bi-compartimental model for linear and non-linear regression analyses. It is shown that hMSCs from UCB do not behave as perfect osmometers. It is worth noting that, the preliminary results of our ongoing research were presented at the *ICheaP12* (*International Conference on Chemical & Process Engineering*, May 2015), and published as conference proceedings in [18]. Accordingly, those results are not reported here, even though for a thorough discussion on the osmotic behavior of hMSCs from UCB they will be necessarily referred to in the sequel. In this paper, the set of experimental runs already shown in [18] was repeated to confirm the unexpected measurements and completed by analyzing the restoration of isotonic conditions after equilibration with hypertonic solutions of permeant and impermeant solute. This new set of experimental runs allows one to fully identify and characterize a peculiar osmotic behavior never reported so far in the literature of cryopreservation: the occurrence of hysteretic phenomena in cell volume excursions depending on the number of osmotic cycles. This result certainly helps the discussion and gives a hint for a modeling interpretation.

## Experimental outline

### Cord blood collection, and isolation and culture of hMSC

Informed, written consent was obtained from anonymous mothers. Comitato Etico of the Azienda Ospedaliera Brotzu AOB (Cagliari, Italy) granted permission to treat the human cord blood samples. The “Banca del Cordone Ombelicale” (Azienda Ospedaliera Brotzu AOB, Cagliari, Italy) provided 15 UCB units. Processing was performed within 15 h since birth time.

After 1:1 blood dilution with phosphate-buffered saline (PBS)/2 mM EDTA, mononuclear cells (MNCs) were isolated by density gradient centrifugation at 400 *g* and 20°C for 40 minutes using a Ficoll-Paque PLUS (GE Healthcare). Once removed from the interphase and washed two to three times with PBS/EDTA, MNCs were suspended in a proliferation medium consisting of minimum essential medium alpha-modification (α-MEM, Sigma-Aldrich) containing 20% fetal bovine serum (FBS, Sigma-Aldrich), 100 U/ml penicillin and 100 μg/ml streptomycin (Sigma-Aldrich), and 2mM L-glutamine (Sigma-Aldrich). Cell counting was performed using an automated cell analyzer (Coulter Counter Multisizer 4, Beckman Coulter). First, cells were seeded at a density of 1×10^6^ cells/cm^2^ in culture dishes (Falcon) and maintained at 37°C in a humidified atmosphere containing 5% CO_2_. After 24 hours of incubation, non-adherent cells were removed and fresh medium was added to dishes as described in the literature [13]. Then, dexamethasone (10^−7^ M) (Sigma-Aldrich) was added in the primary culture medium for a week to reduce the adhesion of monocytes to dishes. One week later, non-adherent cells were removed by replacing the culture medium, and remaining cells were fed weekly with culture medium without dexamethasone [19]. Culture dishes were screened daily to detect developing colonies of adherent cells. After 16 to 20 days from initial plating, fibroblastoid cells were harvested by 0.25% trypsin-EDTA (Sigma-Aldrich) and seeded at a density of 5×10^3^ cells/cm^2^ in a proliferation medium supplemented with 4 ng/ml FGF-Basic (Life Technologies). Attained the 70% to 80% confluence, hMSCs were either sub-cultured or frozen in liquid nitrogen. In this latter case, cells were harvested in trypsin-EDTA, centrifuged at 400 *g* for 5 minutes and suspended at a density of 1×10^6^/ml in the freezing medium (90%vol. FBS and 10%vol. DMSO). Cells were cooled in an isopropanol chamber at 1°C/min down to –80°C, kept at this temperature overnight, and finally transferred in liquid nitrogen.

### hMSCs characterization: Immunophenotypic and differentiation analyses

For surface marker analysis, adherent cells from second to third passages were trypsinized, washed, suspended in 0,1% BSA/PBS (Bovine Serum Albumin by Sigma-Aldrich; Phosphate Buffer Saline), and incubated with the CD105, CD44, CD90, CD34 and CD45 (Millipore), CD73 (BD Pharmingen) mouse anti-human primary antibodies. After 20 min incubation in the presence of the primary antibody at room temperature in the dark, cells were washed three times with 0.1% BSA/PBS and incubated with the secondary antibody (Alexa Fluor^®^ 488 F(ab’)2 fragment of goat anti-mouse IgG (H+L), Life Thechnologies), again for 20 minutes at room temperature in the dark. Then, cells were washed, suspended in a solution 0.1% BSA/PBS and 1% paraformaldehyde, and stored at 4°C until analysis by flow cytometry. Overall, 10000 labeled cells were analyzed using a FACScan flow cytometer running CellQuest software (Becton Dickinson).

To test their potential plasticity, hMSCs from UCB at passages 4 to 6 were subjected to adipogenic, osteogenic, and chondrogenic differentiation. Cells were grown with specific differentiation media. Negative control tests were performed on cells cultured with proliferation medium. The medium was changed every 3 to 4 days, and cultures were fixed at different intervals of time. More specifically, osteogenic differentiation was promoted by seeding the cells in 24-well plates (Costar) at a density of 1×10^4^ cells/cm2 with StemPro Osteogenesis Differentiation Kit (Gibco by Life Thechnologies) for 9 days. The cells were fixed for 10 minutes at 4°C in ice-cold acetone, washed, and incubated with 1% silver nitrate (Carlo Erba) for 45 min under ultraviolet light. Then, the cells were incubated for 5 min with 3% sodium thiosulfate (Carlo Erba), washed, and counterstained with 0,2% safranin (Sigma-Aldrich) stain. To promote adipogenic differentiation, cells were seeded in 24-well plates (Costar) at a density of 5×10^3^ cells/cm2 with StemPro Adipogenesis Differentiation Kit (Gibco by Life Thechnologies) for 21 days. Cells were fixed with 4% paraformaldehyde for 60 min, washed, and stained for 15 min with a working 7 solution of isopropanol 0.5% of Oil Red O (Sigma-Aldrich), filtered and diluted to 60% in distilled H2O. Finally, cells were washed and counterstained with hematoxylin for 1 min. For chondrogenic differentiation, 2.5×10^5^ cells were centrifuged in a 15-ml polypropylene tube, and the pellets were cultured in StemPro Chondrogenesis Differentiation Kit (Gibco by Life Thechnologies) for 21 days. The pellets were fixed with 4% paraformaldehyde for 60 min, embedded in paraffin, cut into 5 μm sections, stained with 1% Alcian blue (Sigma Aldrich) in 3% acetic acid and counterstained with a commercial nuclear fast red solution (Sigma Aldrich).

### Osmotic runs

Isotonic hMSCs were equilibrated at different temperatures in hypertonic solutions consisting of sucrose or DMSO (Sigma-Aldrich) added to PBS (300 mOsm/L). The restoration of isotonic conditions after pre-equilibration with hypertonic sucrose or DMSO solutions was also performed at different temperatures. A freezing-point-depression osmometer (Advanced Micro Osmometer Model 3300, Advanced Instruments, Norwood, MA) was used to measure solution osmolality. Separate experimental runs were performed for individual donors. Cells at passages 3 to 5 were used indifferently. Cell concentration ranged from 2000 to 10000 cells/ml. Since the volume ratio between cells and suspending solution is very small under such circumstances, the osmotic response of cells cannot affect significantly the extra-cellular compartment composition. Every single run was repeated at least three times for all UCB units.

### Apparatus and operating conditions

Cell size was measured using the Coulter Counter Multisizer 4 (Beckman Coulter). Before each experiment, the instrument electrolyte solution was replaced by the appropriate hypertonic solution to avoid mismatch with the sample solution and consequent electrical conductivity gradients [20]. Before any experimental run, the instrument was calibrated using latex beads (diameter 10 μm, Beckman Coulter).

Experimental runs involved both equilibrium and transient conditions. In the former case, cell volumes were measured only after the equilibration time of 5 min, which was estimated in preliminary experiments monitoring the evolution of the cell volume distribution with time. To obtain the Boyle-van’t Hoff (BVH) plot, equilibrium runs were carried out at 23°C and cells were injected into hypertonic solutions of 400, 500, 560, 600 and 900 mOsm/L osmolarities. In contrast, dynamic runs were performed at 17, 27 and 37°C with cells injected into a hypertonic solution with 600 or 2000 mOsm/L osmolarity depending on the use of non-permeant sucrose and permeant DMSO respectively. Cells equilibrated in these hypertonic solutions at 27°C were also exposed back to isotonic PBS at the same three different temperatures to measure their dynamic response.

Additional dynamic runs were performed at 27°C with hypertonic solutions obtained adding DMSO to PBS isotonic solutions, which attained 1000, 1500 and 2365 mOsm/L osmolarity.

**Fig 1:**
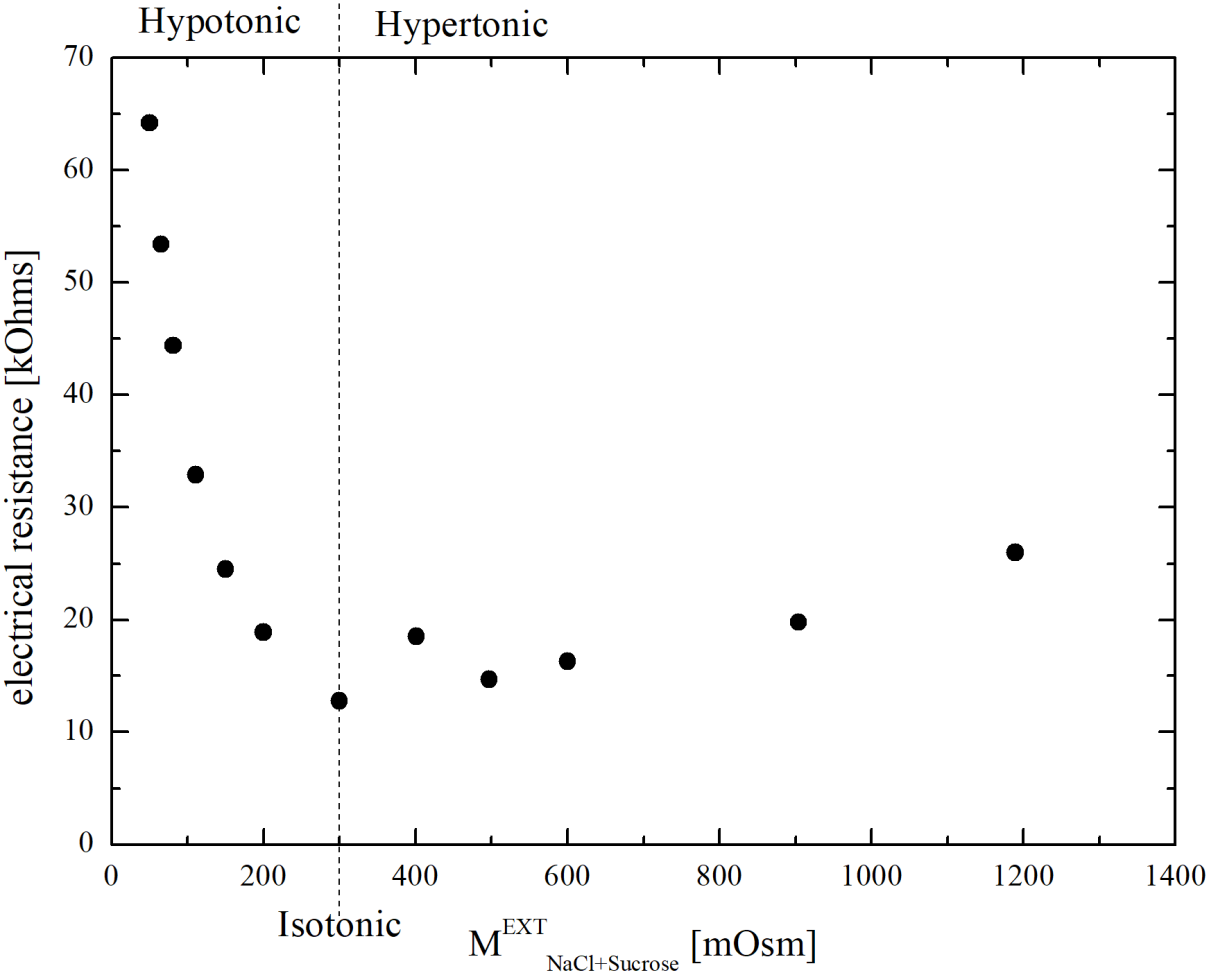
Electrical resistivity (measured by the Coulter Counter) of the suspending solutions in the hypotonic (diluted PBS by distilled water) and hypertonic (sucrose in PBS) range.

Osmotic response under hypotonic conditions was not investigated. Indeed, preliminary experiments performed injecting isotonic cells into PBS solutions diluted by distilled water, i.e. at lower ionic strengths than PBS, pointed out a significant influence of electrolytic composition, which can be seen in Fig 1. Literature has already discussed this Coulter Counter limitation [21,22]. In particular, the possible generation of enhanced electric fields in the Coulter Counter sensing zone in the presence of hypotonic solutions, i.e. at low ionic strengths, can induce the dielectric breakdown of cell membranes, resulting in the underestimation of cell size [23,24].

During osmotic runs, temperature was controlled within a ± 0.2°C interval by circulating water-NaCl bath at 2% wt./vol as suggested by literature [14]. To this aim, isotonic cells were injected into a suitably manufactured beaker allowing perfect mixing, and dispersed in the suspending solution.

### Data treatment

Coulter Counter’s ability of measuring the osmotic response of a relatively large cell population represents a significant advantage over micrographic analysis and direct microscopic inspection, which are necessarily restricted to small samples. In this regard, Coulter Counter’s performances are comparable with those of cytofluorimeters [21]. However, since impedance measurements do not discriminate single cells by debris or cells agglomerates, debris (small volumes) and agglomerates (large volumes) lying outside the interval between minimum *V*_*MIN*_ (*t*) and maximum *V*_*MAX*_ (*t*) volumes of the corresponding cell volume distribution were filtered out.

**Fig 2:**
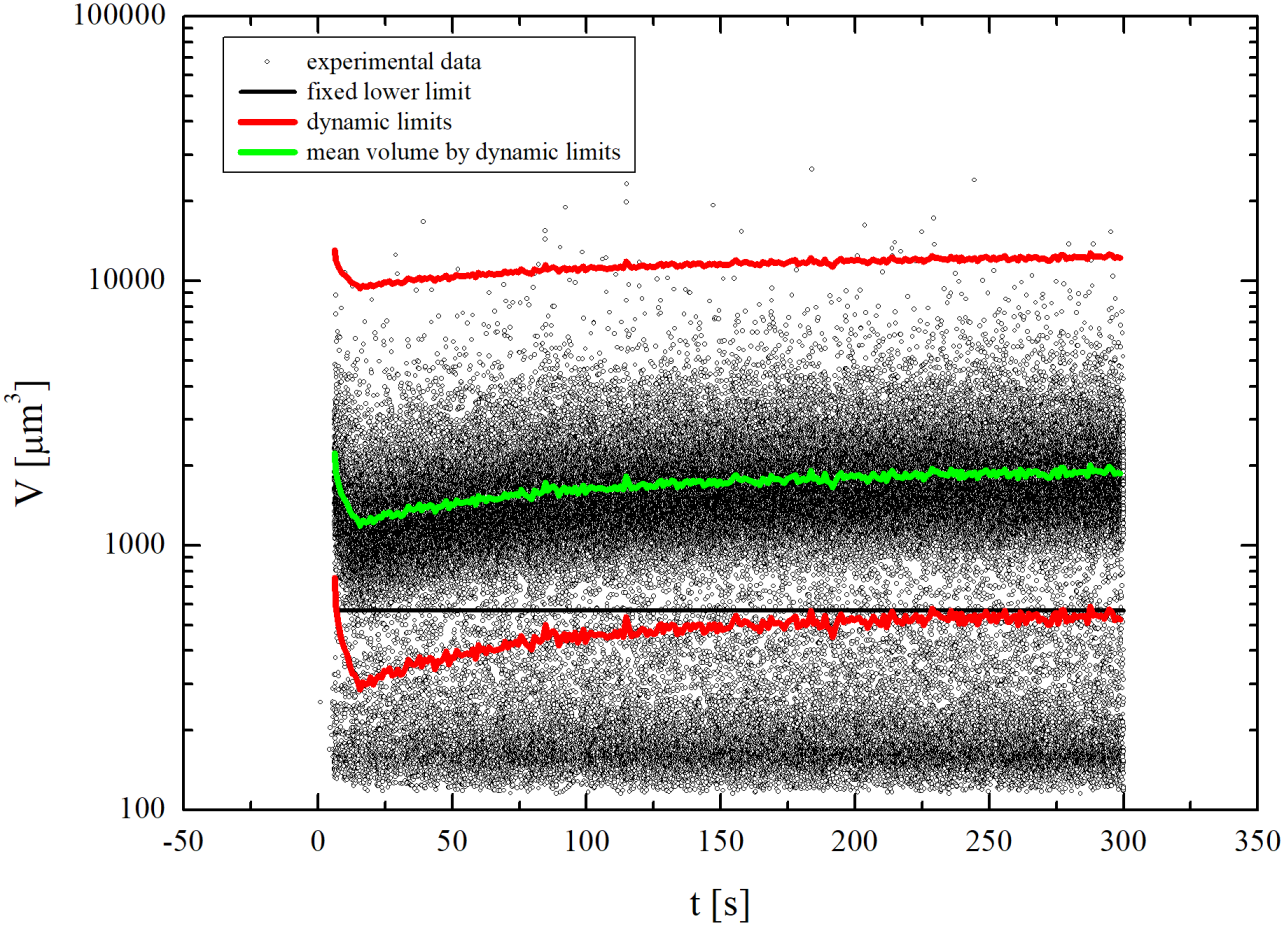
Data treatment on the cells volume measured by the Coulter Counter.

Data in Fig 2 provide a representative case referring to a dynamic run with isotonic cells exposed to a DMSO hypertonic solution. Individual points correspond to the volume and acquisition time measured for any single event detected by the Coulter Counter, i.e. cells, debris and agglomerates. The initial time for the measurements of cell volumes during the dynamic runs was carefully identified by checking the count rate after cells injection according to literature [25]. Once set the dynamic volumic range, the current mean cell volume *V*_*MEAN*_ (*t*), corresponding to the green line in Fig 2, was determined. First, a lower threshold, marked by the black line, was chosen. All measured small-volume events below such threshold were ascribed to debris and no longer taken into account. Second, *V*_*MEAN*_ (*t*) was obtained by averaging every 2 s all the measured events above the threshold. Then, *V*_*MIN*_ (*t*) and *V*_*MAX*_ (*t*) were calculated using the expression

$$V_{MAX;\;MIN}\left(t+1\right)=V_{MAX;\;MIN}\left(t\right)+\frac{A_{MAX;\;MIN}\left(t\right)}{A_{MEAN}\left(t\right)}\;\left[V_{MEAN}\left(t+1\right)-V_{MEAN}\left(t\right)\right]$$ (1)

starting from the maximum and minimum volumes of the corresponding cell volume distribution obtained by separate measurements at isotonic conditions. Here, *A* represents the cell membrane surface area, which is assumed to envelope a spherical volume, i.e. $A={\left(4\pi \right)}^{\frac{1}{3}}\;{\left(3V\right)}^{\frac{2}{3}}$. Finally, the dynamic profile of *V*_*MEAN*_ (*t*) was calculated by averaging every 2 s all the measured events falling inside the dynamic range bounded by *V*_*MIN*_ (*t*) and *V*_*MAX*_ (*t*). Overall, this methodology allows a more reliable data treatment compared with data truncation of fines or deconvolution of measured distributions [26,27].

It is worth noting that the Coulter Counter’s data acquisition frequency of 0.5 Hz translates into cell volume distributions evaluated over about 700 cells under dynamic conditions. This number represents the best compromise between resolution of cell volume distributions and low coincidence factor in the Coulter Counter measurements. Under equilibrium conditions, cell volume distributions were evaluated, instead, over about 2000 cells.

## Modeling

Within the simplest modeling scenario underlying cryopreservation studies, cells are typically regarded as spherical drops of NaCl ideal aqueous solution representing cytoplasm. Being proteins, organelles, and other macromolecules suspended in the cytosol not involved in osmosis, their volumes are grouped generically into the inactive cell volume *V*_*b*_. The inactive cell volume fraction $v_{b}=\frac{V_{b}}{V_{0}}$(i.e. the fraction of inactive cell volume and isotonic cell volume) is assumed to keep constant during the osmotic response. Thus, it is characteristic of the cellular lineage. An oil semi-permeable membrane separates cytosol and inactive volume from external solution. The cell membrane exhibits low resistance to water transport, whereas specific solutes may cross it depending on size, electric charge and hydrogen bonding features. Usually, sucrose and NaCl are assumed to be non-permeant. For this reason, the content of NaCl in intra- and extra-cellular compartments does not change with time. In contrast, permeant CPAs as DMSO may cross the cell membrane as water molecules do, although at slower velocities.

Within this framework, the total cell volume can be written as *V*_*cell*_ = *V*_*w*_ + *V*_*DMSO*_ + *V*_*NaCl*_ +*V*_*b*_, where the inactive cell volume and the NaCl one, *V*_*NaCl*_, remain constant during the osmotic run. In contrast, water (*V*_*w*_) and DMSO (*V*_*DMSO*_) intracellular volumes vary with time in response to the driving forces arising in connection with solute concentration differences between extra- and intra-cellular compartments. The 2-parameter model allows describing the dynamic response of individual cells as a function of such driving forces [28]. In particular, the cell osmotic behavior is accounted for by the equations and corresponding initial conditions below:

$$\frac{dV_{w}}{dt}=-{\text{L}}_{P}\text{ R T A }\left[\left(M_{NaCl+Sucrose}^{ext}-M_{NaCl}^{int}\right)+\left(M_{DMSO}^{ext}-M_{DMSO}^{int}\right)\right]\;;\;t=0\;V_{w}=V_{w}^{iso};$$ (2)

$$\frac{dV_{DMSO}}{dt}={\tilde{\upsilon }}_{DMSO}\;P_{DMSO}\;A\;\left(M_{DMSO}^{ext}-M_{DMSO}^{int}\right)\;;\;t=0\;V_{DMSO}=0,$$ (3)

where *R* is the universal gas constant, *T* the system temperature, *A* the cell membrane area, and ${\tilde{\upsilon }}_{DMSO}$ the DMSO molar volume, equal to 7.1×10^−5^ m^3^/mol. The water and DMSO permeabilities, respectively L_*P*_ and *P*_*DMSO*_, exhibit an Arrhenius-like dependence from temperature. Accordingly, ${\text{L}}_{P}=L_{p}^{0}\;exp\left(-\frac{E_{w}}{R\;T}\right)$ and ${\text{P}}_{DMSO}=P_{DMSO}^{0}\;exp\left(-\frac{E_{DMSO}}{R\;T}\right)$. Similar to inactive volume fraction *υ*_*b*_, they are characteristic of the cellular lineage and must be regarded as adjustable parameters. Hence, the name of the 2-parameter model [28]. For this model, membrane permeation by water and DMSO is governed by the different concentration of impermeant, NaCl and sucrose, and permeant, DMSO, solutes in intra- and extra-cellular compartments. For cells initially at isotonic conditions with no intracellular DMSO, the variation of intracellular osmolalities $M_{NaCl}^{int}=M_{NaCl}^{iso}\;\left(\frac{1-\upsilon_{b}}{\frac{V_{cell}}{V_{cell}^{iso}}-\upsilon_{b}-\frac{V_{DMSO}}{V_{cell}^{iso}}}\right)$ and $M_{DMSO}^{int}=\frac{1}{{\tilde{\upsilon }}_{DMSO}}\;\left(\frac{\frac{V_{DMSO}}{V_{cell}^{iso}}}{\frac{V_{cell}}{V_{cell}^{iso}}-\upsilon_{b}-\frac{V_{DMSO}}{V_{cell}^{iso}}}\right)$ during the osmotic run tends to make the differences with extra-cellular counterparts $M_{NaCl+Suscrose}^{ext}$ and $M_{DMSO}^{ext}$ vanishing. In the absence of differences, intra- and extra-cellular compartments reach the osmotic equilibrium, which involves the conditions

$$\frac{{V_{cell}|}_{equil}}{V_{cell}^{iso}}=\upsilon_{b}+\;\left(1-\upsilon_{b}\right)\;\frac{M_{NaCl}^{iso}}{M_{NaCl+Sucrose}^{ext}}\;\left(1+{\tilde{\upsilon }}_{DMSO}M_{DMSO}^{ext}\right);$$ (4)

$$\frac{{V_{DMSO}|}_{equil}}{V_{cell}^{iso}}={\tilde{\upsilon }}_{DMSO}\;\frac{M_{DMSO}^{ext}}{1+{\tilde{\upsilon }}_{DMSO}\;M_{DMSO}^{ext}}\;\left(\frac{{V_{cell}|}_{equil}}{V_{cell}^{iso}}-\upsilon_{b}\right).$$ (5)

**Fig 3:**
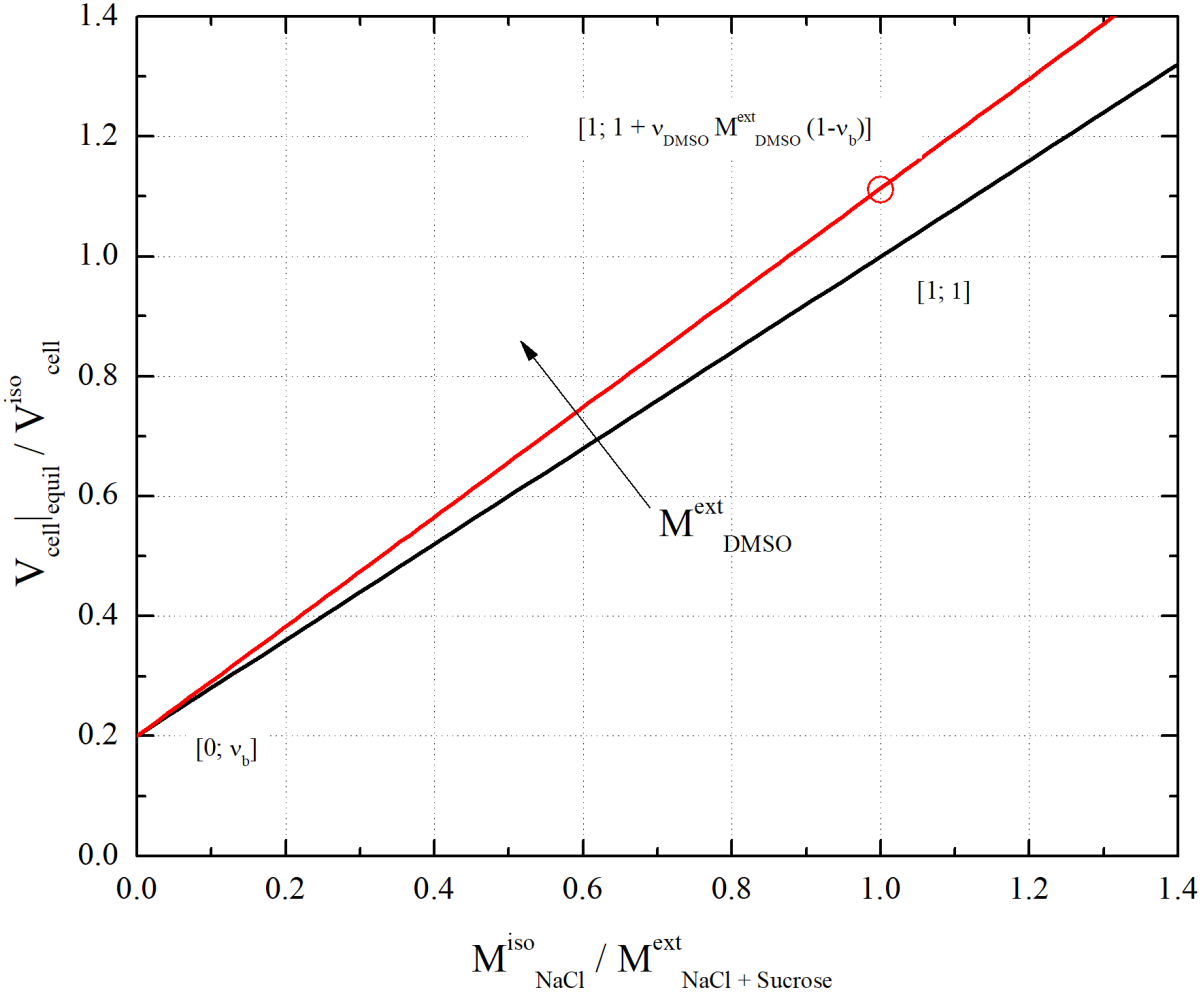
Plot of Eq 4 for the case of *v*_*b*_ = 0.2: black line is the Boyle Van’t Hoff plot (i.e. $M_{DMSO}^{ext}=0$); red line corresponds to the addition of about 10%vol. of DMSO ($M_{DMSO}^{ext}=2,000$ mOsm/L).

According to Eq 4, in the absence of DMSO the so-called BVH plot obtained by showing $\frac{{V_{cell}|}_{equil}}{V_{cell}^{iso}}$ as a function of $\frac{M_{NaCl}^{iso}}{M_{NaCl+Sucrose}^{ext}}$ is linear, with intercept *υ*_*b*_ and slope (1 − *υ*_*b*_). As shown in Fig 3, the line connects the points $\left(\frac{M_{NaCl}^{iso}}{M_{NaCl+Sucrose}^{ext}}=0\;;\;\frac{{V_{cell}|}_{equil}}{V_{cell}^{iso}}=\upsilon_{b}\right)$ and $\left(\frac{M_{NaCl}^{iso}}{M_{NaCl+Sucrose}^{ext}}=1\;;\;\frac{{V_{cell}|}_{equil}}{V_{cell}^{iso}}=1\right)$ [29,30], involving for cells the perfect osmotic behavior.

Eq 4 also predicts that, in the presence of permeant DMSO, the BVH plot keeps linear. The line has still intercept *υ*_*b*_, but the slope increases with $M_{DMSO}^{ext}$. As shown in Fig 3, the line passes through the point $\left(\frac{M_{NaCl}^{iso}}{M_{NaCl+Sucrose}^{ext}}=1\;;\;\frac{{V_{cell}|}_{equil}}{V_{cell}^{iso}}=1+{\tilde{\upsilon }}_{DMSO}\;M_{DMSO}^{ext}\;\left(1-\upsilon_{b}\right)\right)$. This indicates that DMSO addition to isotonic PBS results in the ├ *V*_*cell*_ |_*equil*_ increase. Thus, perfect osmometer cells undergo swelling upon DMSO addition. Overall, permeant DMSO simply adds to the cell volume. In this regard, the 2-parameter model predictions do not differ from the 3-parameter model ones [31].

The derivation of Eq 1 deserves a final note. In this respect, it is worth noting that equations similar to Eq 2 can be written for the total volume of cells belonging to the maximum, mean and minimum cell size classes. Eq 1 can be obtained by dividing the equation relative to the maximum or minimum volume by the one relative to the mean volume, while assuming the factor connected with driving forces constant, keeping the temporal dependence of the geometric factor, and approximating time derivatives by the explicit Euler backward differentiation formula. Since Eq 2 satisfactorily describes the volume excursion of cells belonging to the maximum and minimum size classes, Eq 1 should also allow a reliable estimate of the temporal profile of cell volume during osmotic runs.

## Results and discussion

The isolation of hMSCs by plastic adherence was successful only for 3 UCB units out of the 15 ones available. The 20% success rate is consistent with literature data [32], and can be expected to depend mostly on time elapsed from birth to isolation and size of UCB units [2,13].

**Fig 4:**
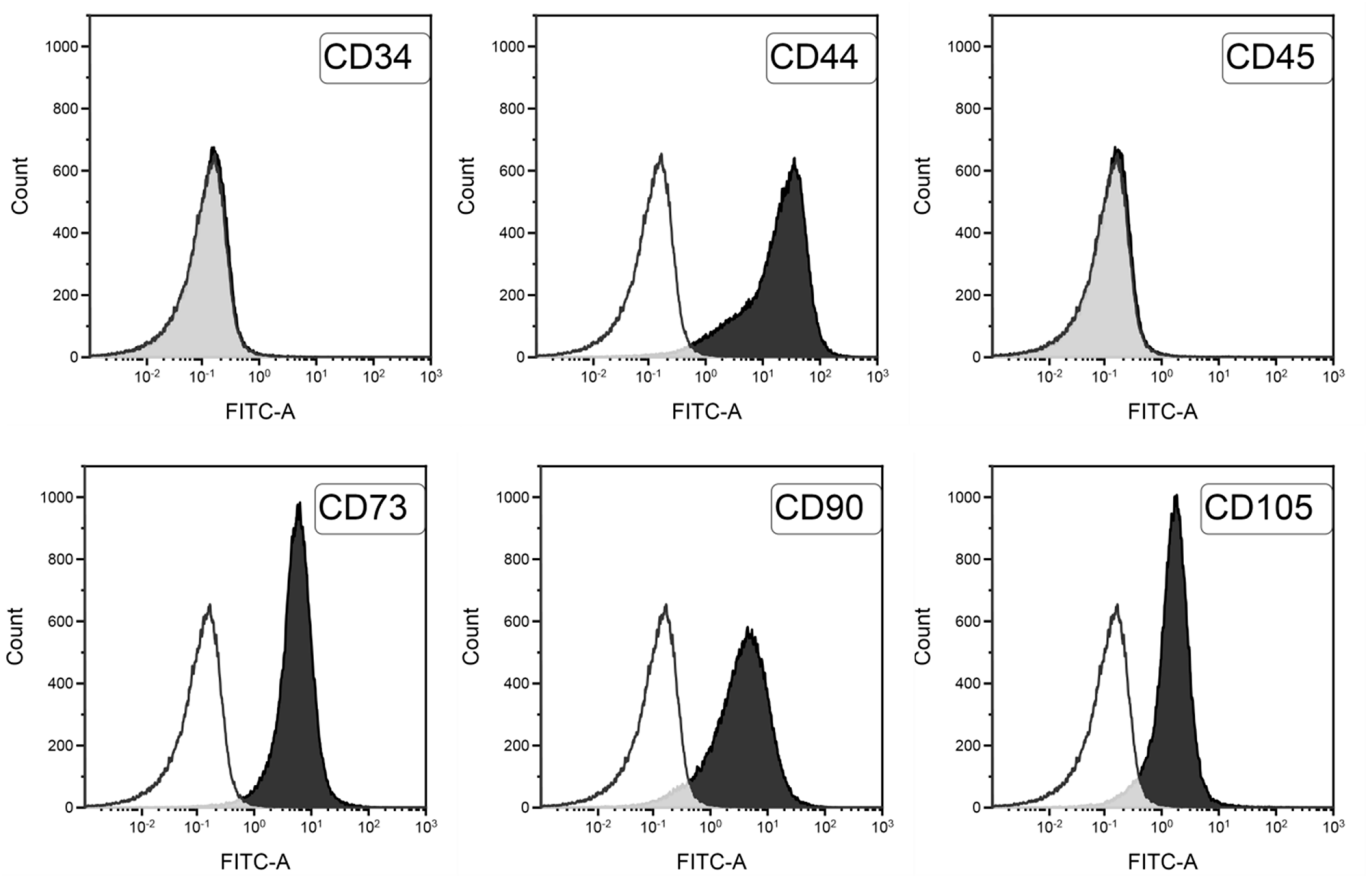
Flow cytometric characterization of isolated hMSCs from UCB. White histograms represent the negative controls, black histograms represent labelled cells.

The phenotypic cytofluorimetric analysis yields similar results for all isolated cells. Data shown in Fig 4 reveal the strong expression of typical markers of mesenchymal line (CD105 91% average, 10% standard deviation; CD90 94% average, 8% standard deviation; CD73 98% average, 1.5% standard deviation; CD44 97% average, 1.8% standard deviation), whereas hematopoietic markers (CD34 0.15% average, 0.01% standard deviation; CD45 0.2% average, 0.15% standard deviation) are not detected.

**Fig 5:**
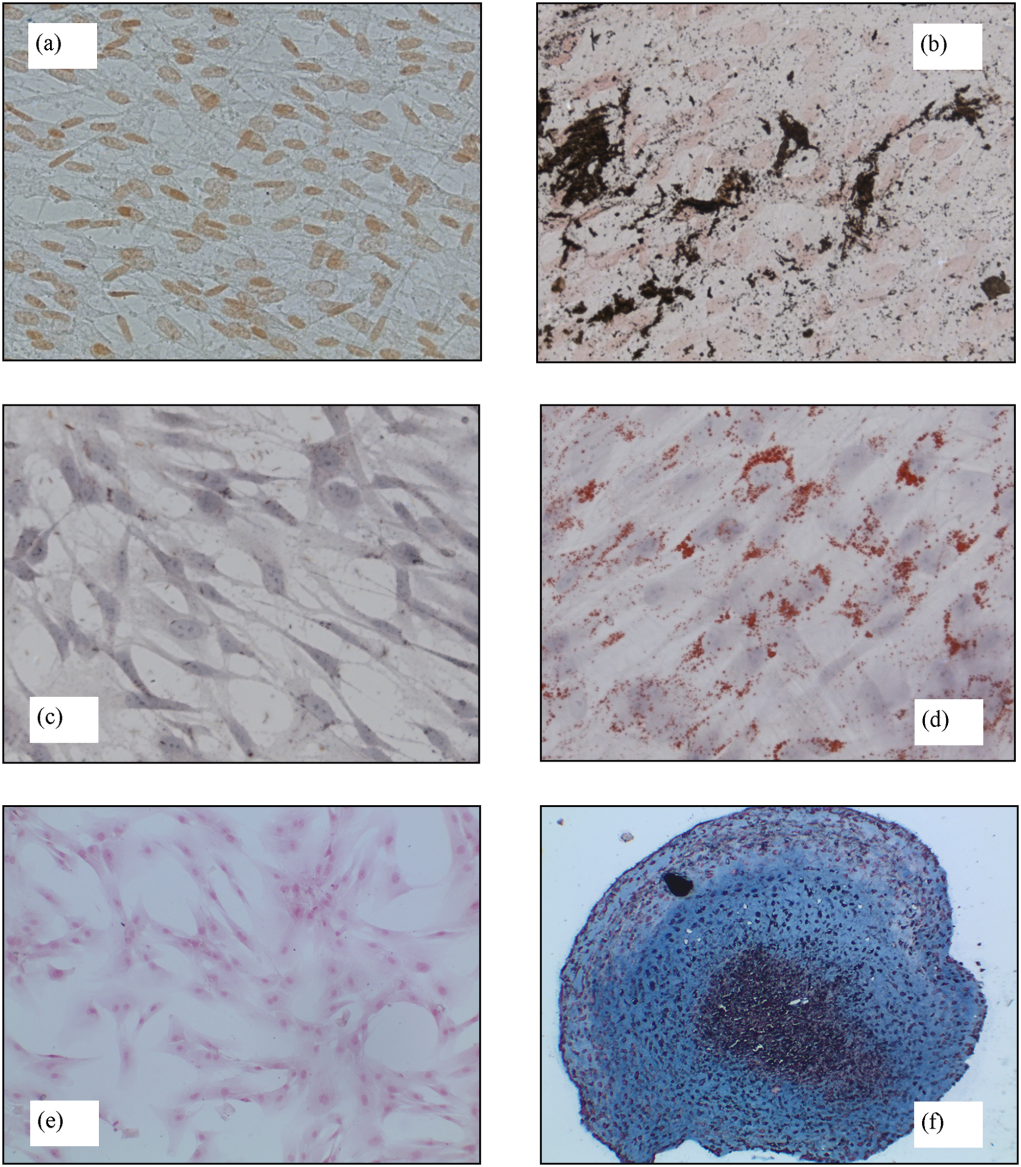
Capability differentiation of isolated hMSCs from UCB: von Kossa staining for osteogenic differentiation in untreated/control (a) and treated cells (b); Oil red O staining for adipogenic differentiation in untreated/control (c) and treated cells (d). Magnification 500x.; Alcian Blue staining for chondrocyte differentiation in untreated/control (e) and treated cells (f). Magnification 200x.

Osteogenic capability was demonstrated by the accumulation of mineralized calcium phosphate according to the von Kossa staining method. Compared with the negative control shown in Fig 5a, the cells successfully isolated from UCB units exhibit a definite osteogenic capability (cf. Fig 5b). The cells also exhibit adipogenic capability. Data plotted in Fig 5d indicate, indeed, the intracellular accumulation of lipid droplets stained with Oil Red O, whereas the negative control in Fig 5c shows no activity. Chondrogenic differentiation was demonstrated by the proteoglycan accumulation stained with Alcian Blue: compared with the negative control shown in Fig 5e, the cells exhibit a definite chondrogenic capability (cf. Fig 5f). All the successfully isolated cells from the UCB units exhibit the same behaviour.

The osmotic behavior of hMSCs from UCB was investigated under equilibrium conditions at room temperature injecting isotonic cells into different hypertonic solutions. Experimental findings where $\frac{{V_{cell}|}_{equil}}{V_{cell}^{iso}}$ is shown as a function of $\frac{M_{NaCl}^{iso}}{M_{NaCl+Sucrose}^{ext}}$ to obtain a BVH plot are given in [18]. Since hMSCs from the 3 different UCB units have similar behavior, only the arithmetic mean of cell volumes was considered. Data gave rise to a linear arrangement with positive slope. Therefore, under hypertonic conditions hMSCs from UCB seemingly behave as perfect osmometers.

Best fitting the experimental points allows estimating the inactive volume fraction *υ*_*b*_ to a remarkable accuracy degree, its best-fitted value being equal to about 0.2 (quite different from the only one reported in the literature for the same cells [2] obtained using mannose as non-permeant solute, and analysing cells under a microscope). This value was utilized to determine *L*_*P*_ and its Arrhenius dependence on temperature. To this aim, dynamic runs involving the injection of isotonic cells in a hypertonic solution obtained adding sucrose to PBS were performed at three different temperatures. The obtained experimental data where the cell volume normalized to its isotonic value is shown as a function of time are reported in [18]: the exposure of cells to the hypertonic solution invariably induces shrinkage. The highest the temperature, the highest the rate of volume variation. Therefore, cells experiencing the highest temperature are those reaching first the new equilibrium condition. The model equations allow reproducing quite well the experimental behaviour, providing reliable estimates of water permeability *L*_*P*_ at the three different temperatures investigated.

**Fig 6:**
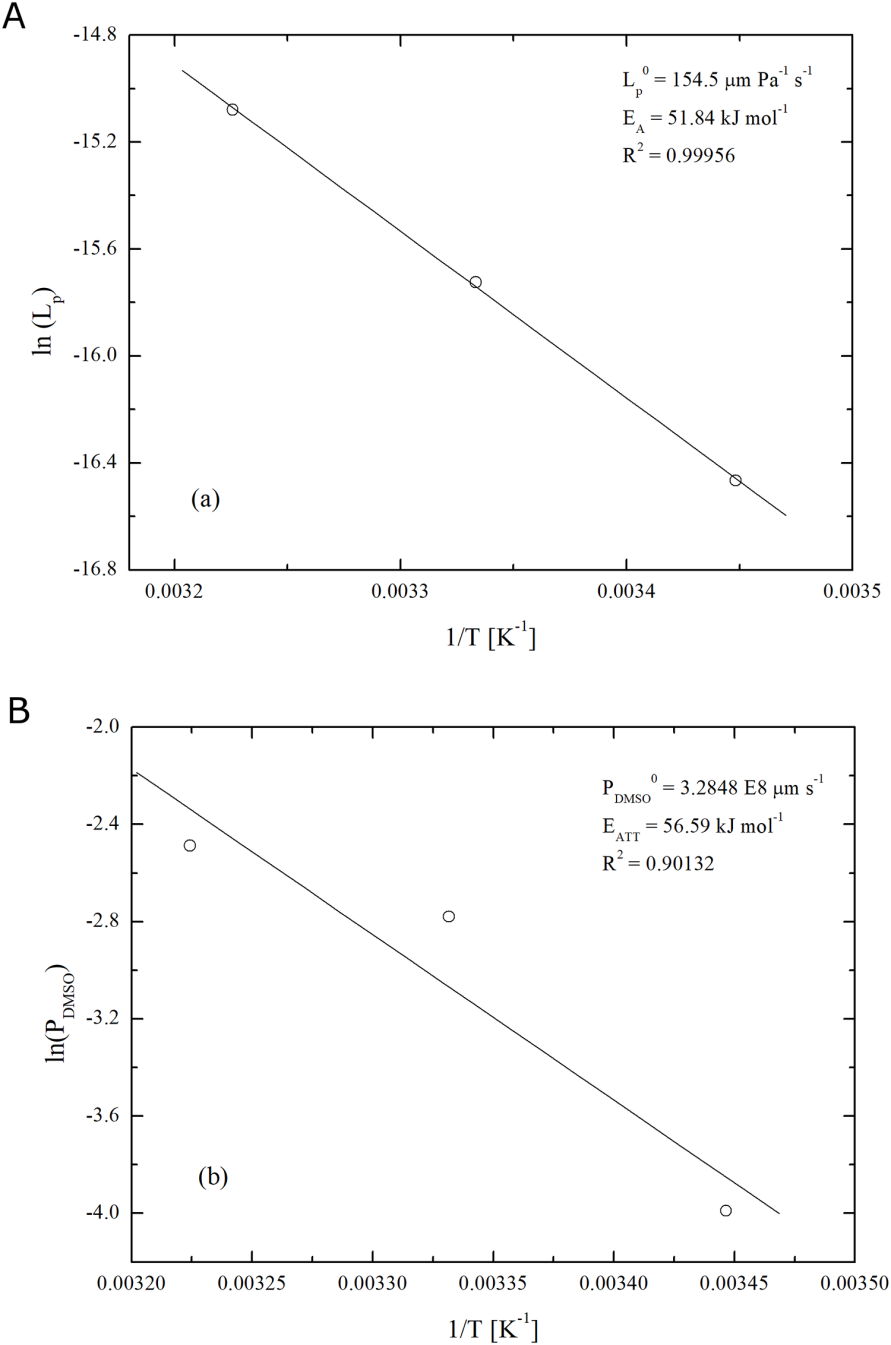
Arrhenius dependence of water permeability L_*P*_ (a) and DMSO permeability *P*_*DMSO*_ (b) for hMSCs from UCB.

As shown in Fig 6a, the three *L*_*P*_ values give rise to a reliable Arrhenius plot, thus enabling the estimate of the pre-exponential factor $L_{P}^{0}$, equal to about 154.5 μm/(Pa s), and the apparent activation energy *E*_*w*_, approximately equal to 51.8 kJ/mol. To the best of authors’ knowledge, this osmotic transport parameter has never been determined before for this cell line while it falls into the relatively broad range of values reported in the literature for different cell lineages [25,33].

To determine the membrane permeability to DMSO, *P*_*DMSO*_, and the Arrhenius parameters governing its thermal variation, isotonic cells were injected in the hypertonic solution obtained adding DMSO to PBS. The experimental results where normalised cell volume is plotted as a function of time are reported in [18]. Experiments were performed at the same temperatures set for dynamics runs aimed at evaluating water permeability. Due to the enhanced permeability of water relative to DMSO, the cell volume first decreases and then increases, the shrink-swell dynamics being faster at higher temperature.

Although the observed behaviour is in line with previous experimental observations for other cell lineages [25,33–36], the model equations are no longer able to reproduce satisfactorily the experimental data if the inactive volume, *υ*_*b*_, and the water permeability, *L*_*P*_, are given the values determined in independent experiments as discussed above. Whereas model predictions are still acceptable in the initial stage of volume shrinkage, no match with experimental points is observed in the second stage. In this case, the model first underestimates and then overestimates the mean cell volume, which involves overestimating also the final equilibrium volume.

The incapability of the model of reproducing the experimental behaviour suggests the activation of one, or more, cellular mechanisms to limit volume excursion during the swelling phase. Consequently, it raises a crucial issue for modelling addressing the osmotic behaviour of hMSCs. Quite surprisingly, it seems that the observed behaviour has never been reported in the literature heretofore. This can be ascribed to the attention paid almost exclusively to dynamic experiments in the presence of CPAs only [25,33,34,36–39]. Accordingly, inactive volume fraction is determined through a BVH plot, and water and CPA permeabilities are evaluated simultaneously following CPA addition. In no case, independent experiments are performed to determine water permeability separately from CPA one. There is only one study adopting the ideal best fitting procedure [35] to reliably evaluate the adjustable model parameters in a sequential fashion. However, it does not discuss in detail the final stage of dynamic runs involving CPA addition, as in all the other references. Hence, the idea that cells invariably behave as perfect osmometers.

However, this is not the case under investigation. Experimental data clearly indicate a deviation from the ideal behaviour. Further clue to non-ideality comes from the evidence that modelling equations are able to reproduce the experimental behaviour only if inactive volume fraction, *υ*_*b*_, is allowed to vary with temperature. Under such condition, model predictions match experimental values almost perfectly which allows estimating *P*_*DMSO*_ by best fitting. The resulting values are shown in Fig 6b. Such *P*_*DMSO*_ estimates exhibit a reasonable Arrhenius dependence on temperature, with the pre-exponential factor, $P_{DMSO}^{0}$, equal to about 3.3×10^8^ μm/s and the apparent activation energy, *E*_*DMSO*_, around 56.6 kJ/mol. The variation of inactive volume fraction, *υ*_*b*_, with temperature is reported in [18].

The latter evidence is quite unexpected based on the available literature, although a *υ*_*b*_ increase in the presence of DMSO was already observed and ascribed to a barrier contrasting the water flux involving DMSO itself [40]. To investigate the possible role of DMSO, additional osmotic experiments were performed at 27°C using solutions with different DMSO concentration. At any given DMSO osmolality, $M_{DMSO}^{ext}$, model equations were used to interpolate experimental points and estimate *υ*_*b*_ by best fitting. To such aim, the *L*_*P*_ and *P*_*DMSO*_ were calculated starting from their Arrhenius parameters. As reported in [18], *υ*_*b*_ increases with $M_{DMSO}^{ext}$, from about 0.2 to 0.6. The significant data scattering does not allow clarifying the functional dependence.

**Fig 7:**
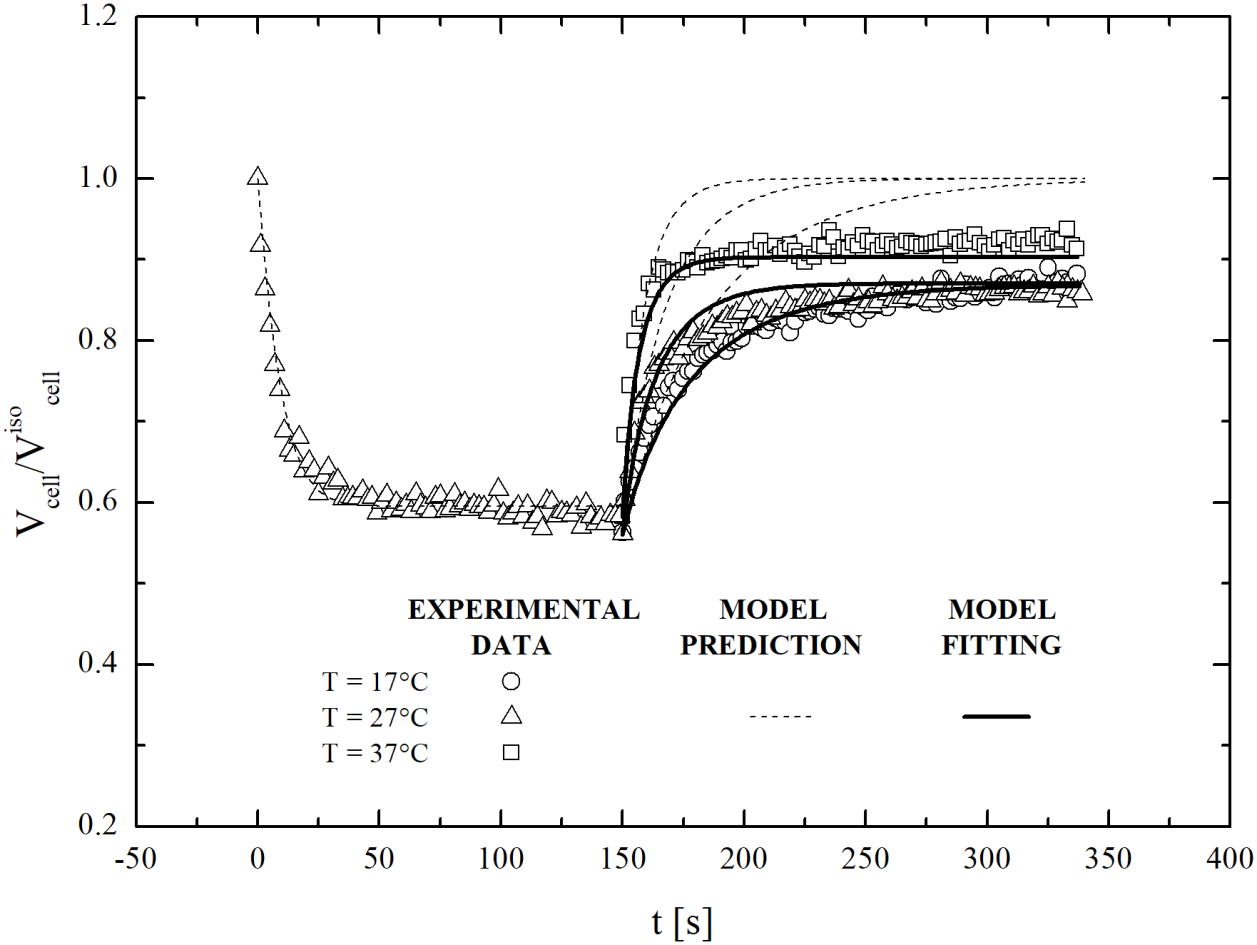
Dynamic swelling of dehydrated cells (previously equilibrated with non-permeant solute, $M_{Sucrose+NaCl}^{ext}=600$ mOsm/L) contacted back to isotonic conditions at 17, 27 and 37°C: experimental data and model fittings.

Additional experiments were performed under dynamic conditions to investigate swelling further. In particular, restoration of isotonic conditions was studied at three different temperatures using an aqueous solution of sucrose at 600 mOsm/L. Obtained results are shown in Fig 7. It can be seen that cell volume does not return to the initial isotonic value: rather, it equilibrates at a smaller value. This demonstrates that the observed regulatory response of hMSCs from UCB does not depend specifically on the presence of DMSO. In fact, it seems to be characteristic of any possible swelling excursion.

**Fig 8:**
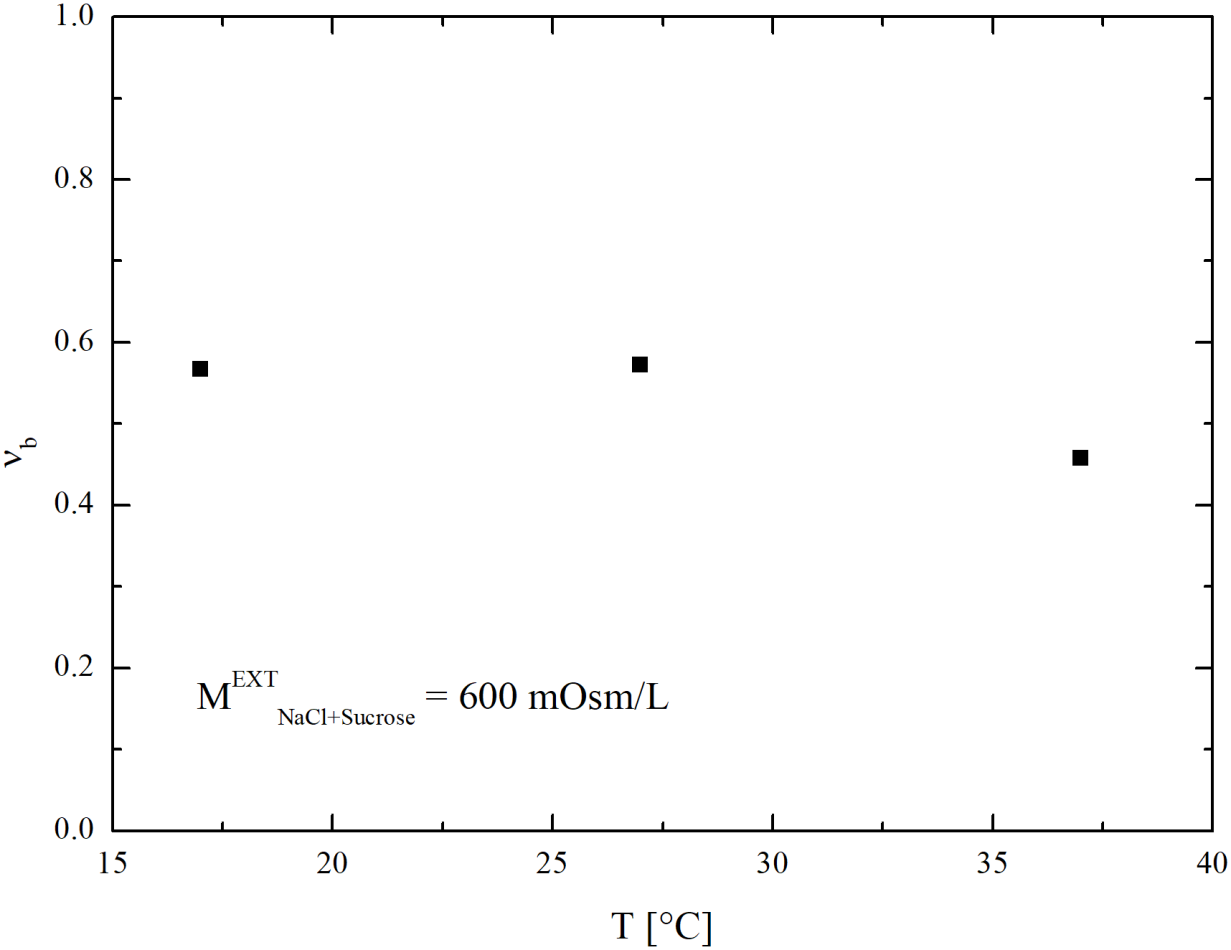
Temperature dependence of osmotic inactive cell volume fraction of hSMCs from UCB obtained at constant sucrose concentration from the fitting of data reported in [18].

As in the case of DMSO, model equations reproduce experimental data only if inactive volume fraction, *υ*_*b*_, is left free to vary with temperature. Best-fitted *υ*_*b*_ values are shown in Fig 8 as a function of temperature. Overall, they are comparable with those obtained for DMSO and reported in [18].

**Fig 9:**
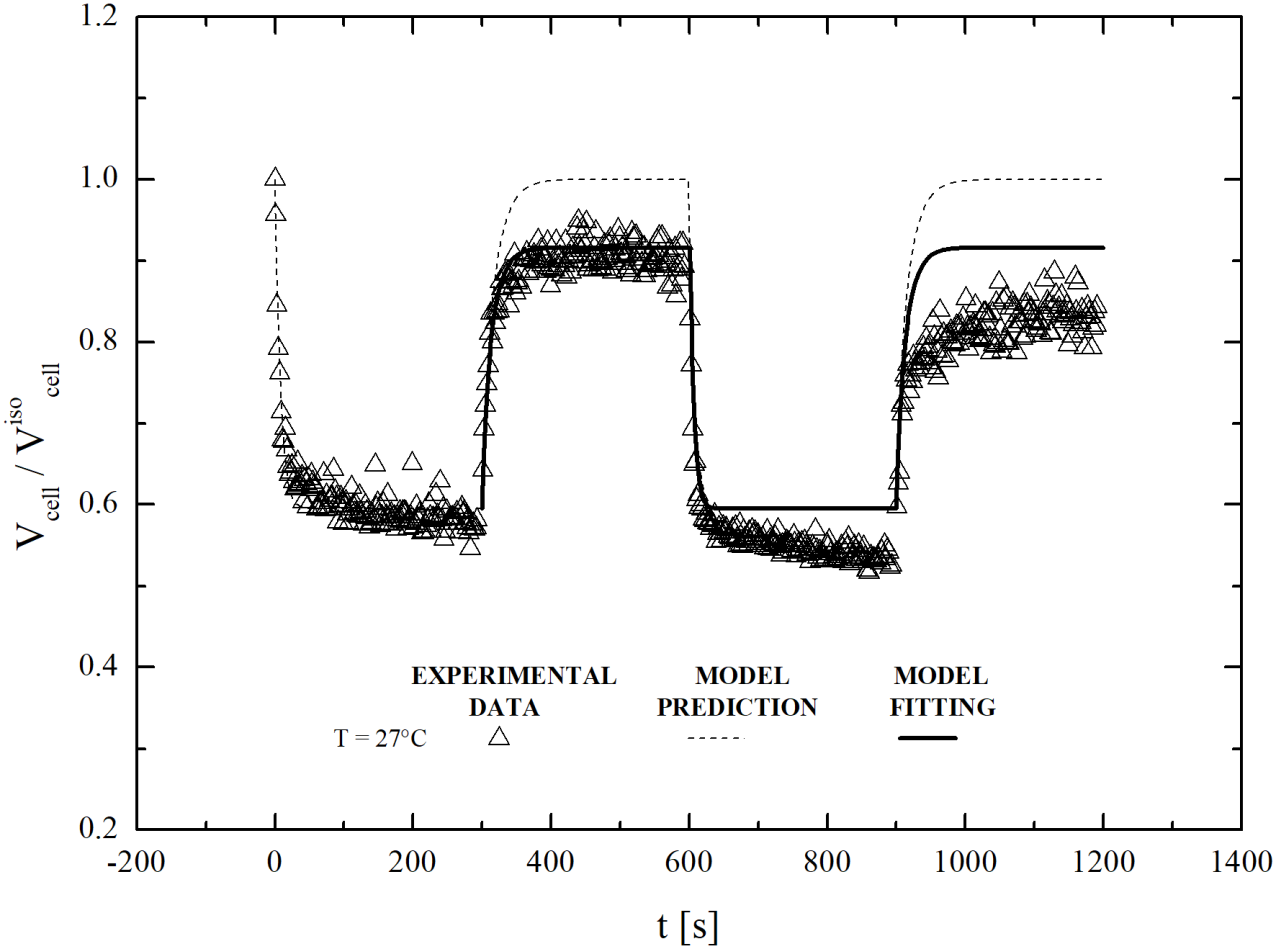
Two-cycle dynamic swelling of dehydrated cells (after equilibration with non-permeant solute, $M_{Sucrose+NaCl}^{ext}=600$ mOsm/L) contacted back to isotonic conditions at 17°C: Experimental data, model prediction and model fitting.

Interestingly, a sequential increase of the equilibrated *υ*_*b*_ value is observed in cells subjected to two consecutive osmotic cycles involving the exposure to sucrose (600 mOsm/L) and the restoration of isotonic conditions. As shown in Fig 9, the equilibrated cell volume during the second osmotic cycle tends to attain a value smaller than the one reached during the first osmotic cycle. Similar to previous cases, model equations reproduce the swelling behaviour only if *υ*_*b*_ can vary freely. Accordingly, the *υ*_*b*_ value increases from 0.2 to 0.46 for the shrinkage and swelling phases of the first osmotic cycle. However, this adjustment does not allows one to follow the shrinkage and swelling phases of the second osmotic cycle, and a new, higher value for the inactive cell volume fraction should now be used.

Above-mentioned empirical evidence definitely highlights the unexpected osmotic behaviour of hMSCs from UCB, which raises serious questions concerning the concept of isotonic volume. This latter quantity seemingly depends on the osmotic history of the cell, being affected by the number of shrink-swell cycles as well as by temperature and DMSO content. In addition, the 2-parameter model is clearly unable to describe satisfactorily the osmotic response of hMSCs from UCB.

**Fig 10:**
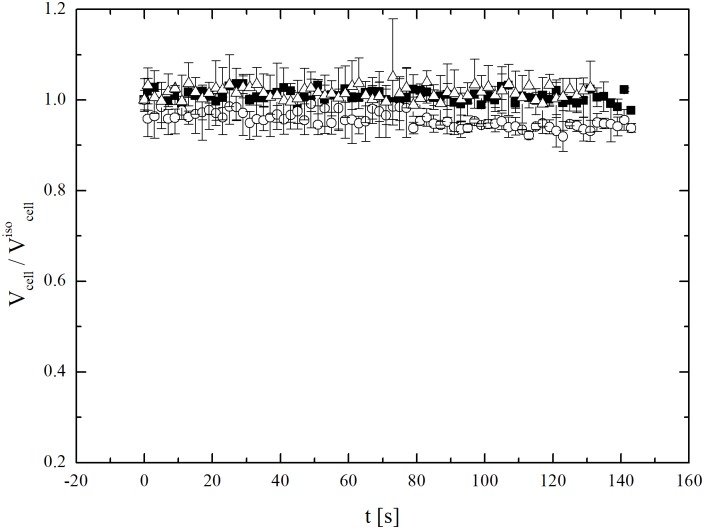
Osmotic response under isotonic condition at 27°C (along with experimental errors) of hMSCs from UCB previously subjected to different osmotic stresses and then re-plated and grown for two days. The data refers to the cells of three different donors, separately analysed.

To make the scenario more complex, it appears that the inability of cells subjected to different osmotic stress of attaining the initial isotonic volume is only temporary. Indeed, additional experiments on cells subjected to osmotic stresses, re-plated, and grown for two days reach the isotonic volume when exposed to an isotonic solution. As shown in Fig 10, not only they reach the isotonic volume (about 1800 μm^3^), but also seem able to keep it indefinitely. This means that hMSCs become able again to attain the expected isotonic volume after two-day cultivation under ideal conditions. Two days represent a relatively long time interval if compared with characteristic time scales typically associated with osmosis during cryopreservation.

**Fig 11:**
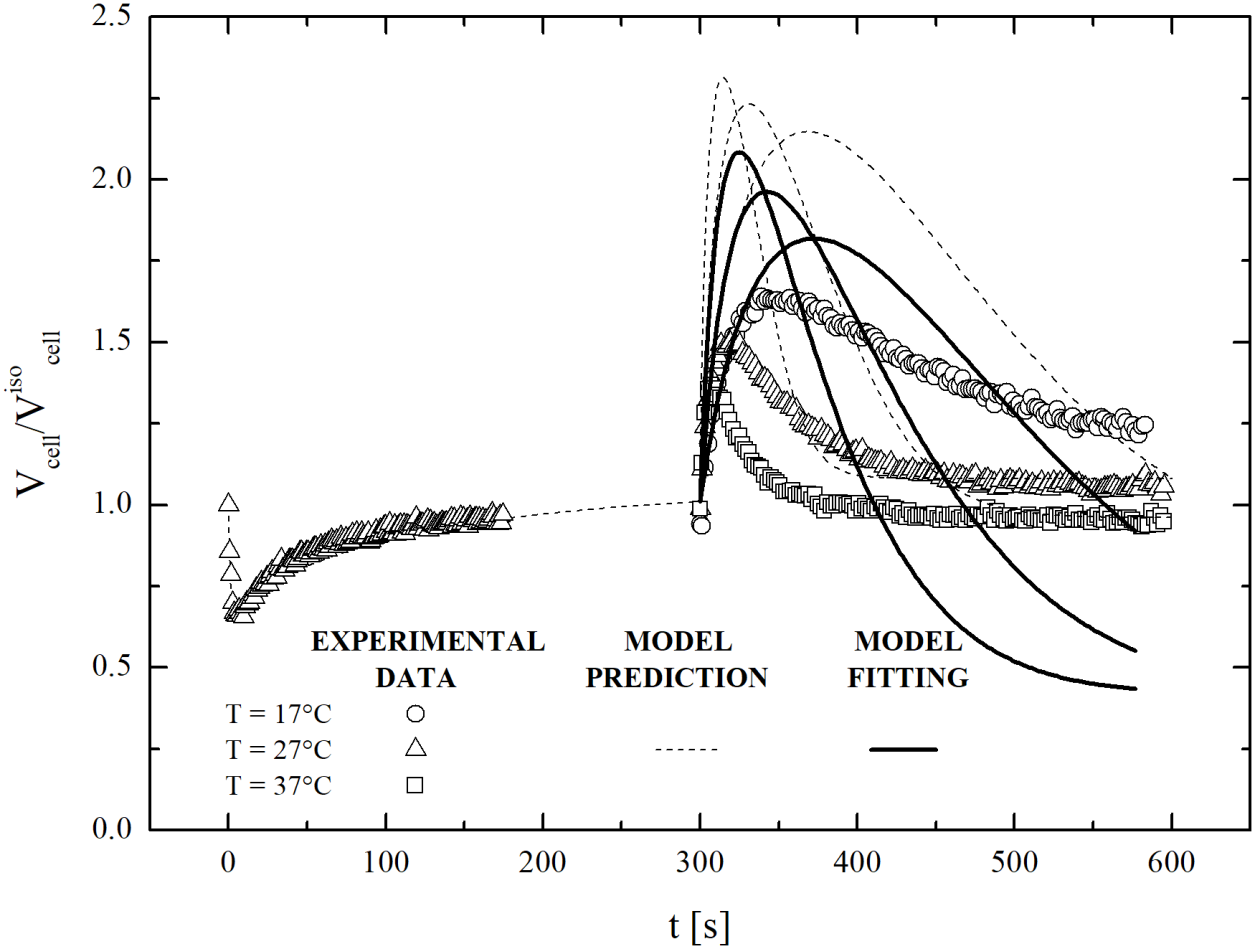
Dynamic removal of CPA from cells (previously equilibrated at 27°C with DMSO in PBS, $M_{NaCl}^{ext}=300$ mOsm/L and $M_{DMSO}^{ext}=1,700$ mOsm/L) contacted back to isotonic conditions at 17, 27 and 37°C: Experimental data with model fittings and predictions.

The dynamic removal of DMSO at the three temperatures exposing cells to isotonic PBS was also investigated. The results obtained for cells previously equilibrated with 2000 mOsm/L DMSO at 27°C are shown in Fig 11. Once more, the 2-parameter model is unable to reproduce the experimental behaviour. This strongly suggests that hMSCs from UCB can activate specific volume regulation mechanisms.

## Conclusions and future directions

To optimize a cryopreservation protocol a number of operating conditions needs to be specified: cooling/thawing rates, cryo-protectant addition/removal strategy, and system size and geometry. Indeed, after a cryopreservation protocol cells fate in terms of post-thaw viability and functionality largely depends on osmotic rate, ice formation and disappearance, CPA cytotoxicity and excessive volumic excursions: all these phenomena are influenced by the operating conditions listed above and vary from cell to cell lineage. Unfortunately, even for a single cell line the number of experiments increases so much that, only a sequential optimization may be typically adopted, thus allowing one to reach only a pseudo-optimal condition. In this context, it is widely acknowledged that modeling represent a valid alternative to account for the effect of more than one parameter at time [10,11]. To this aim, this work addresses the accurate study of the osmotic behavior of hMSCs from UCB.

This study raises two fundamental issues concerning the cell response to osmotic stress and the reliability of the 2-parameter model in describing it satisfactorily. The variation of inactive volume with temperature and number of osmotic cycles is the crucial evidence questioning current knowledge about osmotic processes involving hMSCs and cells in general. Marking the incapability of hMSCs from UCB to restore the equilibrium volume at isotonic conditions after osmotic stress, the variation of inactive volume also highlights the inadequacy of the 2-parameter model in representing the kinetic features underlying volume changes in these specific cells subjected to osmosis.

Particularly interesting is the sensitivity of inactive volume to the number of osmotic cycles. Never attempted before, the exposure of cells to two consecutive hypertonic-to-isotonic condition stages clearly demonstrates that hysteretic phenomena affect the cell response to osmosis. Although temporary, such phenomena take place on time scales relevant to cryopreservation. Therefore, experiments and models need accounting for them properly.

Far from being explanatory, the above-mentioned conceptual framework suggests the activation of control mechanisms for cell volume excursions not considered in the 2-parameter model. Borrowing from available literature, intracellular mechanisms involving ion-pumps and/or ion-channels could represent a possible candidate for explaining the observed osmotic behaviour [41–43]. However, it is worth noting that ion pumps work on relatively long time scales, not compatible with the rapid response to osmosis exhibited by hMSCs. Therefore, other mechanistic scenarios could be invoked. Starting from the experimental evidence, the control mechanism acting on hMSCs from UCB is switched on during any swelling phase, and prevents the reaching of the initial isotonic volume, when the cells are exposed back to isotonic conditions. To account for this lack of cell volume at the new equilibrium condition, it can be hypothesised that intracellular salt is actually a permeant solute. Then, by removing the fundamental assumption of the 2-parameter model, when a cell swells its membrane tension increases, and specific mechano-sensitive ion-channels on the membrane open [44], so that intra-cellular salt may be exchanged with the environment. This would explain the lack of cell volume at the new equilibrium condition after swelling. The mechanical relaxation of cellular membranes (that follows in order to maintain homeostasis) reduces membrane tension, thus closing the mechano-sensitive ion channels and permitting the cells to reach a new, lower, isotonic volume. This temporary character of mechanical relaxation during any osmotic stress, when membrane tension varies to eventually reach its resting value, should account also for the hysteretic phenomena reported as depending on the number of the osmotic cycles.

## Supporting information

S1 File Editable file (Origin software v8 by OriginLab Corp.) for Fig 1 in the paper. (OPJ)

S2 File Editable file (Origin software v8 by OriginLab Corp.) for Fig 2 in the paper. (OPJ)

S3 File Editable file (Origin software v8 by OriginLab Corp.) for Fig 3 in the paper. (OPJ)

S4 File Editable file (Origin software v8 by OriginLab Corp.) for Fig 6a in the paper. (OPJ)

S5 File Editable file (Origin software v8 by OriginLab Corp.) for Fig 6b in the paper. (OPJ)

S6 File Editable file (Origin software v8 by OriginLab Corp.) for Fig 7 in the paper. (OPJ)

S7 File Editable file (Origin software v8 by OriginLab Corp.) for Fig 8 in the paper. (OPJ)

S8 File Editable file (Origin software v8 by OriginLab Corp.) for Fig 9 in the paper. (OPJ)

S9 File Editable file (Origin software v8 by OriginLab Corp.) for Fig 10 in the paper. (OPJ)

S10 File Editable file (Origin software v8 by OriginLab Corp.) for Fig 11 in the paper. (OPJ)

S11 File Editable file (Excel by Microsoft Office Professional 2010) for the statistical analysis of CD markers expression. (XLSX)

S12 File PDF file of the research ethics approval (Italian language). (PDF)
