# Supplementary material for: Osmotic behaviour of human mesenchymal stem cells: Implications for cryopreservation
Source: PLoS One. 2017 Sep 8;12(9):e0184180. doi: 10.1371/journal.pone.0184180 (PMC5590898; doi:10.1371/journal.pone.0184180)
Supplement: S12 File — (PDF) [file pone.0184180.s012.pdf]

Prot._78_del____28/02/2013_______

COMITATO ETICO INDIPENDENTE DELL'AZIENDA OSPEDALIERA "G. BROTZU"
VERBALE n.__2/2013____ RIUNIONE DEL 27.02.2013

"Analisi sperimentale e modellistica della crioconservazione di cellule staminali cordonali"

Sperimentatore: **Dr. Marino Argiolas , Dr. Alberto Cincotti**
Reparto di sperimentazione: Banca del Cordone Ombelicale/Università di Cagliari
Sponsor**: spontaneo**
Documentazione esaminata:

1. Lettera di intenti del 30.11.2012 pervenuta il 06.12.2012;
2. Protocollo;
3. Informativa e Consenso;
4. Sinossi.

Vista la richiesta di effettuare lo studio sotto la responsabilità del Dr. Argiolas e del Dr. Cincotti;
Valutato il protocollo sia dal punto di vista della validità scientifica sia per quanto riguarda la correttezza dal punto di vista etico;
Valutata l'Informativa al paziente e Consenso informato da sottoporre ai soggetti che partecipano alla sperimentazione ritenuta "una responsabile informativa";
Valutato che la ricerca risulta corrispondere alle regole di buona pratica clinica di cui al Decreto del Ministero della Sanità del 15.07.1997 relativo al "Recepimento delle linee guida dell'Unione europea di buona pratica clinica per l'esecuzione delle sperimentazioni cliniche dei medicinali";
Verificata la completezza della documentazione presentata,

IL COMITATO APPROVA ALL'UNANIMITA'

Il parere sopra espresso si intende limitato esclusivamente alle versioni citate in oggetto ed alla documentazione presentata ed espressamente citata. Ogni variazione allo stesso deve obbligatoriamente essere sottoposta al parere di questo C.E., così come previsto dalle vigenti norme nazionali ed europee. Tutte le segnalazioni relative ad eventi avversi seri e reazioni avverse serie e/o inattese, la conclusione dello studio ed ogni eventuale sua integrazione dovrà essere comunicata allo scrivente C.E.. **Lo studio potrà essere intrapreso solo a seguito della autorizzazione del Direttore Generale formalizzato da apposito atto deliberativo**. Ogni eventuale sospensione o motivata interruzione dello studio dovrà essere comunicato al Direttore Generale ed al Comitato Etico. Lo sperimentatore è tenuto ad interpellare in qualsiasi momento il C.E. ogni qual volta si renda necessaria una nuova valutazione etica. **Lo sperimentatore è tenuto ad informare il C.E. sull'andamento della sperimentazione almeno una volta l'anno**, con relazione scritta riportante il numero dei casi arruolati.

La Segreteria
Dr.ssa Fiorella Garofalo
Fiorella Garofalo

REGIONE AUTONOMA DELLA SARDEGNA
Azienda OspedalieraBrotzu

COMITATO ETICO INDIPENDENTE DELL'AZIENDA OSPEDALIERA "G. BROTZU"
RIUNIONE DEL 27.02.2013

Presidente
Dr.ssa Andreina Farris
(Vice prefetto Cagliari)

Vice Presidente
Dr Paolo Congiu
(Clinico)

Prof. Giuseppe Binaghi
(Clinico)

Dr. Carlo Follesa
(Esperto bioetica)

Dr. ssa Silvana Maniscalco ASSENTE
(Rappresentante volontariato)

Dr.ssa Bruna Dettori
(Responsabile Infermieristico Dip. di Emergenza Azienda Ospedaliera "G. Brotzu")

Dr Luigi Minerba
(Docente di Statistica Medica Biostatistico)

Dr. ssa Michela Pellecchia
(Direttore Farmacia Ospedaliera Azienda Ospedaliera "G. Brotzu")

Dr.ssa Prof. Mariangela Serra
(Farmacologo)

Dr.ssa Marinella Spissu ASSENTE
(Rappresentante della Direzione Sanitaria dell'Azienda Ospedaliera "G. Brotzu")

Dr. Prof. Roberto Targhetta
(Clinico oncoematologia)
